# Cysteine proteases during larval migration and development of helminths in their final host

**DOI:** 10.1371/journal.pntd.0005919

**Published:** 2018-08-23

**Authors:** Alexandra Grote, Conor R. Caffrey, Karina M. Rebello, David Smith, John P. Dalton, Sara Lustigman

**Affiliations:** 1 Center for Genomics and Systems Biology, Department of Biology, New York University, New York, New York, United States of America; 2 Center for Discovery and Innovation in Parasitic Diseases, Skaggs School of Pharmacy and Pharmaceutical Sciences, University of California San Diego, La Jolla, California, United States of America; 3 Laboratório de Toxinologia and Laboratório de Estudos Integrados em Protozoologia, Instituto Oswaldo Cruz, Fiocruz, Rio de Janeiro, RJ, Brazil; 4 School of Biological Sciences, Medical Biology Centre, Queen’s University Belfast, Belfast, Northern Ireland, United Kingdom; 5 Department of Microbiology and Immunology, School of Medicine, University of Michigan, Ann Arbor, Michigan, United States of America; 6 Lindsley F. Kimball Research Institute, New York Blood Center, New York, New York, United States of America; Swiss Tropical and Public Health Institute, SWITZERLAND

## Abstract

Neglected tropical diseases caused by metazoan parasites are major public health concerns, and therefore, new methods for their control and elimination are needed. Research over the last 25 years has revealed the vital contribution of cysteine proteases to invasion of and migration by (larval) helminth parasites through host tissues, in addition to their roles in embryogenesis, molting, egg hatching, and yolk degradation. Their central function to maintaining parasite survival in the host has made them prime intervention targets for novel drugs and vaccines. This review focuses on those helminth cysteine proteases that have been functionally characterized during the varied early stages of development in the human host and embryogenesis.

## Cathepsin B- and L-like proteases facilitate invasion of host tissues by larval helminth parasites

The skin and intestinal wall represent physical barriers to pathogen entry into their hosts. In order to successfully breach these barriers, parasites must effectively degrade an array of host proteins. At the same time, parasites need to minimize tissue damage and the induction of innate immune responses in order to quickly and successfully establish infection in the human or animal host. Cysteine proteases of parasitic organisms are the focus of considerable attention, as they are specifically adapted to effectively degrade host tissues to aid penetration and migration.

## Skin penetration by schistosome larvae—A role fulfilled by evolutionarily diverse proteases

Invasive larvae (cercariae) of the schistosome blood fluke must penetrate the epidermis and dermis in order to access the circulatory system and facilitate their establishment in the host. For the cercariae of *Schistosoma japonicum*, a cathepsin B2 cysteine protease is considered the main penetration tool [1], and cathepsin B (CPB) activity has been identified in the cercarial secretions, suggesting that this proteolytic enzyme mediates skin invasion [2]. A comparative study showed that the acetabular gland contents of *S*. *japonicum* cercariae have a 40-fold greater CPB-like activity than those of *S*. *mansoni*, suggesting that CPB is far more relevant to invasion by *S*. *japonicum* [1]. A CPB peptidase has also been identified in the cercariae of the bird schistosome *Trichobilharzia regenti*; it exhibits 77% sequence similarity to the cathepsin B2 in *S*. *mansoni* [3].

The evidence [1] suggesting that a cysteine protease is deployed by the more evolutionarily “ancient” or zoonotic schistosome species (*S*. *japonicum* and *T*. *regenti*) during skin invasion is in striking contrast to the functionally orthologous but evolutionally divergent cercarial elastases. These degradative enzymes are a closely related group of serine proteases that are released during skin penetration by the African schistosomes *S*. *mansoni* and *S*. *haematobium*. The use of cercarial elastases by the these species was proposed as “an unusual biochemical product” [1] compared to other schistosomatids and platyhelminths, and may reflect an adaptation by these parasites to preferentially infect humans without eliciting a potentially parasiticidal inflammatory response [1].

Interestingly, Dresden and coworkers several decades ago identified the presence of biochemically undefined cysteine proteases in schistosome eggs [4]. Recent findings using a functional degradomics strategy on the excretory–secretory products (ESP) of *S*. *mansoni* eggs identified a clan CA cysteine protease with activity at neutral pH [5]. The possible functions of these egg proteases include yolk degradation (as found for similar proteases in insect eggs and in filarial worms, as described above), egg hatching, and/or facilitating the passage of eggs through host tissues [6].

### *Fasciola* species

CPB and cathepsin L (CPL) proteases are secreted by the infective, newly encysted juvenile (NEJ) stage of the liver fluke *Fasciola hepatica* and are crucial to excystation and then penetration by the parasite of the host intestinal wall and liver capsule [7, 8]. RNA interference-mediated (RNAi) silencing of either the NEJ CPB (FhCB) or L (FhCL) in vitro reduced the parasite’s ability to transverse the rat intestinal wall using an ex vivo tissue model [7]. As they penetrate the gut wall, the parasite secretes 3 distinct CPB proteases (FhCB1, FhCB2, and FhCB3), which are down-regulated as the parasite migrates into the liver tissue [9, 10]. Interestingly, a coincident up-regulation of FhCL3 expression occurs as the parasite migrates from the intestine and enters the liver parenchyma [9, 11]. These data suggest a concerted role for the FhCBs and FhCL3 in the early infection stage. Furthermore, asparaginyl endopeptidases (legumains), which are found in abundance in the *F*. *hepatica* NEJs secretome, are likely employed to rapidly process and activate FhCB and FhCL zymogens to functionally mature enzymes [12, 13].

FhCL3 is unique in that its active site is modified for efficient degradation of collagen fibers, a particularly important adaptation to allow the parasite to penetrate the highly collagenous Glisson’s capsule of the liver [5]. Notably, the collagenolytic activity of FhCL3 favors Gly at the P_3_ and Pro at P_2_ positions in its protein or peptide substrate, consistent with the Gly-Pro-X repeat motif found in collagen. By contrast, the FheCL1, secreted by the blood-feeding adult, had a strong preference for P_2_ Leu, Phe, and Ala that fit into the S_2_ pocket of its active site; these amino acids are most predominant in hemoglobin, suggesting a specific adaptation of FhCL1 to the digestion of this major blood protein [14].

Cathepsins B1 (FgCatB1), B2 (FgCatB2), and B3 (FgCatB3) have been identified in various *F*. *gigantica* life stages [15, 16]. FgCatB2 and FgCatB3 are only expressed in *F*. *gigantica* metacercariae and NEJs [15, 16]. The abundance of FgCatB3 in metacercariae suggests that the protein is stored and could also facilitate degradation of the parasite cyst wall once the parasite reaches the duodenum. Since NEJs are considered a nonfeeding life stage, FgCatB2 and FgCatB3 may collectively play a role in parasite invasion and migration across the intestinal wall by degrading connective tissues. Supporting this proposal is the ability of recombinant FgCatB3 to efficiently degrade gelatin and fibronectin [15]. A cDNA encoding FgCatB1 was identified in each life stage associated with the mammalian host, suggesting a general role in proteolytic digestion, although future characterization of functional enzyme is required to elucidate its substrate specificity and biological role [16].

### Opisthorchis

Protease activity studies of ESP of *Opisthorchis viverrini* discovered 1 major cysteine protease (30kDa). The CPL-like protease had an enzymatic profile similar to other CPL proteases from related flukes, including optimal activity at pH 6.0 and inhibition by the cysteine protease inhibitor E-64. Using the fluorogenic peptide substrate Z-Phe-Arg-AMC, most cysteine proteolytic activity was found in the metacercariae, followed by the ESP, egg, and adult worms. Elevated expression of these CPL-like proteases in the metacercariae suggests that they may play a role in larval excystation during mammalian infection [17].

### Paragonimus

Two cysteine proteases (27 and 28 kDa) were detected in the ESP of newly encysted *Paragonimus westermani* metacercariae [18]. These enzymes are involved in metacercarial encystment [18], tissue invasion [19], and immune system evasion [13]. Immunolocalization analysis revealed that both enzymes are present in the excretory bladders of metacercariae [20]. Four CPBs (CsCB1, CsB2, CsB3, and Cs4) were characterized in the *Clonorchis sinensis* life stages [21]. These enzymes are localized in excretory vesicles, oral suckers, and tegument of metacercariae and cercariae [22, 23]. In addition, a CPL was localized in the tegument of both larval stages [24].

## Cysteine proteases in molting and embryogenesis

Phylogenetic analysis of the nematode CPL-like cysteine proteinases using 3 techniques indicates that they form 4 different clades [25]. In *Brugia malayi*, 2 clade I subfamilies of the CPL-like cysteine proteases (*Bm*-CPL) were identified: clade group Ia includes *Bm*-CPL-1, -4, and -5, and clade Ic includes *Bm*-CPL-2, -3, -6, -7, and -8 [25]. The CPL proteases of group Ia as well as cathepsin Z-like (CPZ) proteases have been extensively studied in filarial worms [25–28]. By employing methods to block enzyme function, RNAi, and/or treatment with cysteine protease inhibitors, these proteases were shown to be essential for embryogenesis in *B*. *malayi* female worms [28], as well as for L3 to L4 molting of *Onchocerca volvulus* [29], *B*. *malayi* [30], and *Dirofilaria immitis* [31]. More recently, it was shown that 2 members of the group Ic CPLs might also have a role during symbiosis [32]. Many filarial species harbor an endosymbiotic bacterium of the genus *Wolbachia* [33–37]. As the endosymbiont has limited biosynthetic capabilities, it is plausible that the filarial host supplements *Wolbachia* with amino acids produced by protease degradation of host proteins required for their fitness [38]. Reduction of *Bm-*CPL-3 and *Bm-*CPL-6 transcripts using RNAi caused a significant decrease in *Wolbachia* DNA and a disruption of microfilarial development and release [32].

The functions of filarial CPL-1 and CPZ-1 during embryogenesis are mostly inferred from studying *Ce*-CPL-1, a *Caenorhabditis elegans* CPL-like protease belonging to clade Ib of CPLs [25]. RNAi targeting *B*. *malayi* adult female worms with *Bm-*CPL-1 or *Bm-*CPL-5 dsRNA established that these enzymes are localized in the same tissues in filarial worms as they are in *C*. *elegans* [28, 39]. RNAi with *Ce-*CPL-1 activity resulted in embryonic lethality and a transiently delayed growth of larvae to adults, suggesting an essential role for CPL-1 during embryogenesis and most likely during postembryonic development. Although the precise function of CPL-1 during embryogenesis in filarial worms is not yet clear, it could be involved in regulating the processing of yolk proteins and processing of nutrients responsible for synthesis and/or in the degradation of eggshell (as suggested for the cysteine proteases in schistosome eggs (see above section “Skin penetration by schistosome larvae—A role fulfilled by evolutionarily diverse proteases”).

**Fig 1:**
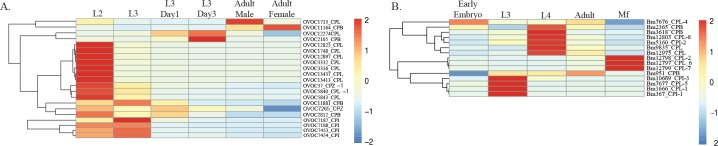
Expression patterns of genes encoding cysteine proteases and their inhibitors over the life cycle of *O*. *volvulus* and *B*. *malayi*. A. Expression of CPL-like, CPZ-like, and CPB-like proteases and their inhibitors over the lifecycle of *O*. *volvulus*. Gene expression in reads per kilobase of transcript per million mapped reads (RPKMs) is z-score normalized and then used to cluster genes based on expression [43]. B. Expression of CPL-like, CPZ-like, and CPB-like proteases and their inhibitors over the lifecycle of *B*. *malayi*. Gene expression in fragments per kilobase of transcript per million mapped reads (FPKMs) is z-score normalized and then used to cluster genes based on expression [45]. As these data were collected as part of 2 independent studies, the units of normalization differ, as do some of the sampled stages. CPL, cathepsin L; CPZ, cathepsin Z; CPB, cathepsin B.

In filarial parasites, the molting of L3 to L4 occurs immediately upon infection of the human host marking the establishment of infection. In several filarial nematodes, this molt depends on the activity of CPL-1 and CPZ-1 [40, 41]. Required for both apolysis and ecdysis, these cathepsins are probably involved in the breakdown of the old cuticle, degradation of cuticle-anchoring proteins, and, potentially, the synthesis of the new cuticle through the processing of proproteins [40]. These proteases are stored in the glandular esophagus of L3 and released during molting [42]. An analysis of the evolutionary history of the filarial nematodes using Ensembl Compara revealed an expansion of CPL-like enzymes in the filarial nematodes as compared to the 3 outgroup species, *Ascaris suum*, *C*. *elegans*, and *Trichuris muris* [43]. Notably, based on annotation and sequence homology (BioProject accession PRJEB513), there appears to be an expansion of the 1a group of CPL-like enzymes in *O*. *volvulus* as compared to *B*. *malayi*, which has 3 group Ia CPL-like proteases, *Bm-*CPL-1, *Bm*-CPL-4, and *Bm*-CPL-5 [43] (Fig 1A). All 7 annotated CPLs in *O*. *volvulus* have the inhibitor domain I29, the presence of which is characteristic of the extended proregions of the Ia group of cysteine proteases. Analysis of the *O*. *volvulus* transcriptome during the parasite life cycle (PRJEB2965) revealed significant differences in the expression of the CPL and CPZ proteases, i.e., a marked up-regulation in the vector-derived L2 stage compared to L3 (Fig 1A) [44]. This suggests that CPLs and CPZs are highly transcribed in the L2s and are then stored in the glandular esophagus of L3 for their known function in the L3 to L4 molt, while also potentially contributing to the molting of L2 to L3. Interestingly, RNAi targeting *Bm-*CPL-1 in *B*. *malayi*-infected mosquitos has verified that CPL-1 is important for the L2 to L3 molt; specifically, it prevented the parasite’s development within the mosquito and inhibited parasite migration inside the mosquito vector [30]. Two CPB-like proteases are also highly expressed in *O*. *volvulus* L2 and are grouped together with the CPLs and CPZs based on their expression profiles (Fig 1A). Although different life stages were sampled, expression data from Choi YJ and colleagues [45] from *B*. *malayi* shows a similar pattern of up-regulation of CPL-like and CPB-like proteases during molting. The orthologues in *B*. *malayi*, Bm3618 and Bm2365, to the 2 highly up-regulated CPB-like proteases during molting in *O*. *volvulus*, OVOC11881 and OVOC2812, are similarly highly up-regulated during molting in *B*. *malayi*.

The function(s) of filarial cysteine proteases during molting are likely regulated by their endogenous cysteine protease inhibitors. In *O*. *volvulus*, CPL-2 (or “onchocystatin”) is localized to the hypodermis and cuticle of the larvae during the L3 to L4 molt, specifically to the region of cuticle separation, where it may regulate the cysteine proteases required for molting [46]. As a result of a recent detailed analysis of the transcriptome and proteome of filarial worms over their various life stages, it is clear that the regulation of expression of cysteine proteases is in concert with other serine, aspartic, and metallo proteases. It will be interesting to determine whether the functions of cysteine proteases during molting, development, embryogenesis, and migration in the invertebrate and human hosts as well as symbiosis are associated with the activities of these other enzymes.

Finally, for comparison, the genome of the dog heartworm *Dirofilaria immitis* contains 10 CPLs and 2 CPZs, as well as 3 cystatins [47], and based on the transcriptome, the highest CPL expression is in L3, which correlates with the expression of the inhibitor cystatin. Expression of CPZ is highest in the microfilaria, suggesting an additional role in the molt within the intermediate vector host [47].

**Key learning points**

- Cysteine proteases are key contributors to the invasion of host tissues by helminth parasites and their various developmental stages.
- The CPL-like (cathepsin L) enzymes are expanded in the genomes of filarial parasites.
- Cysteine proteases in filarial parasites are essential for molting and embryonic development.
- Cysteine proteases may also be involved in maintaining symbiosis in filarial parasites.
- Possible roles for helminth egg cysteine proteases in yolk, egg hatching, and/or for facilitating the passage of eggs through host tissues.

**Top five papers**

1. Dvořák J, Fajtova P, Ulrychova L, Leontovyc A, Rojo-Arreola L, Suzuki BM, et al. Excretion/secretion products from Schistosoma mansoni adults, eggs and schistosomula have unique peptidase specificity profiles. Biochimie. 2016;122:99–109.
2. Dvořák J, Mashiyama ST, Braschi S, Sajid M, Knudsen GM, Hansell E, et al. Differential use of protease families for invasion by schistosome cercariae. Biochimie. 2008;90(2):345–58.
3. Cwiklinski K, Dalton JP, Dufresne PJ, La Course J, Williams DJ, Hodgkinson J, et al. The Fasciola hepatica genome: gene duplication and polymorphism reveals adaptation to the host environment and the capacity for rapid evolution. Genome Biol. 2015;16:71.
4. Lustigman S, Melnikow E, Anand SB, Contreras A, Nandi V, Liu J, et al. Potential involvement of Brugia malayi cysteine proteases in the maintenance of the endosymbiotic relationship with Wolbachia. Int J Parasitol Drugs Drug Resist. 2014;4(3):267–77.
5. Bennuru S, Cotton JA, Ribeiro JM, Grote A, Harsha B, Holroyd N, et al. Stage-Specific Transcriptome and Proteome Analyses of the Filarial Parasite Onchocerca volvulus and Its Wolbachia Endosymbiont. MBio. 2016;7(6).
